# Supplementary material for: N6-Methyladenosine Positively Regulates Coxsackievirus B3 Replication
Source: Viruses. 2024 Sep 11;16(9):1448. doi: 10.3390/v16091448 (PMC11437462; doi:10.3390/v16091448)
Supplement: Supplementary file 1 [file viruses-16-01448-s001.zip › viruses-3166289-supplementary.pdf]

Supplementary Material

Figure S1

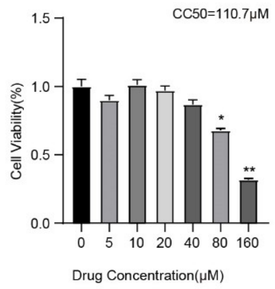

Figure S1. m^{6}A methylation inhibitor 3-DAA inhibits CVB3 replication in HeLa Cells. Cell viability was analyzed using the CCK8 assay after 48 h of 3-DAA treatment.

Figure S2

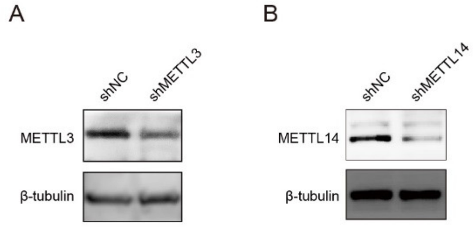

Figure S2. Western blotting was carried out to check the efficiency of knockdown of METTL3 (A) and METTL14 (B) in HeLa cells.

Figure S3

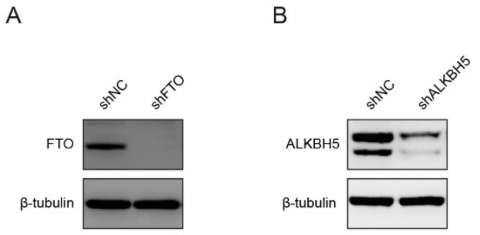

Figure S3. Western blotting was carried out to check the efficiency of knockdown of FTO (A) and ALKBH5 (B) in HeLa cells.

Figure S4

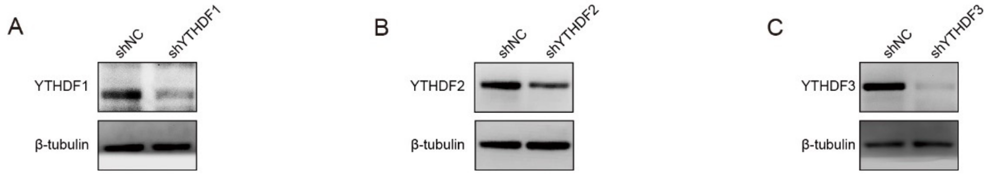

Figure S4. Western blotting was carried out to check the efficiency of knockdown of YTHDF1 (A), YTHDF2 (B) and YTHDF3 in HeLa cells.

Supplementary Table S1.

Prediction of N6-methyladenosine (m6A) modification site for CVB3 RNA

No.	Position	Sequence context	Score(binary)	Score(knn)	Score(spectrum)	Score(combined)	Confidence
1	187	UCAAGCACUUCUGUUACCCCGGACUGAGUAUCAAUAGACUGCUCA	0.725	0.717	0.413	0.600	Moderate
2	202	ACCCCGGACUGAGUAUCAAUAGACUGCUCACGCGGUUGAAGGAGA	0.693	0.865	0.441	0.601	Moderate
3	1532	AGCUCAAGUAUCAACGCAAAAGACUGGGGCACAUGAGACCGGGCU	0.699	0.742	0.595	0.659	High
4	1974	AAGCUGCCUGAUGCUUUGUCGAACUUAGGACUGUUUGGGCAGAAC	0.660	0.473	0.540	0.602	Moderate
5	1981	CUGAUGCUUUGUCGAACUUAGGACUGUUUGGGCAGAACAUGCAGU	0.754	0.687	0.494	0.646	High
6	2021	GCAGUACCACUACUUGGGCCGAACUGGGUAUACCAUACAUGUGCA	0.590	0.462	0.547	0.566	Low

7	2479	CAAUGUGUGCCGAGUACAAUGGACUACGUUUGGCCGGGCACCAGG	0.686	0.619	0.462	0.593	Moderate
8	3411	AGGUCUGAGUCGACCAUAGAGAACUUCCUAUGUAGGUCAGCAUGC	0.591	0.480	0.532	0.561	Low
9	3967	CGUGGAUACCUAGACCACCUAGACUCUGCCAAUACGAGAAGGCAA	0.623	0.668	0.497	0.575	Low
10	3999	UACGAGAAGGCAAAGAACGUGAACUUCCAACCCAGCGGAGUUACC	0.625	0.546	0.523	0.580	Low
11	4518	GCAAUGGAACAGGGAGUGAAGGACUAUGUGGAACAGCUUGGAAAU	0.751	0.720	0.595	0.687	Very high
12	4951	AAAAACACGAAUUCCUGAACAGACUCAAACAGCUCCCCCUGUUAG	0.654	0.538	0.595	0.624	High
13	5693	CAUCCAAUUCAUAGACAGAAGAACUCAAGUCAGAUACUCCCUCGA	0.639	0.606	0.449	0.561	Low
14	5862	CCACCACCACCAGCUAUCGCGGACUUGCUUAAAUCAGUGGAUAGC	0.681	0.835	0.438	0.592	Moderate
15	6337	AUAAGGAUGGUACAAACCUAGAACUGACACUGCUCAAGUUGAACA	0.675	0.746	0.511	0.613	Moderate
16	6922	AGGCUGUUGACCAUUAUGCCGGACAAUUGGCCACCCUAGACAUUA	0.629	0.648	0.495	0.576	Low
17	7083	AUCCUCUCAAAGAAGACCAGGGACCUUACUAAGCUGAAAGAGUGC	0.636	0.528	0.603	0.617	Moderate

18	7156	UGGUAACCUAUGUGAAAGACGAACUCAGAUCUGCAGAGAAGGUGG	0.640	0.650	0.520	0.592	Moderate
19	7221	AUUGAGGCGUCCAGUUUGAAUGACUCUGUGGCAAUGAGACAGACA	0.619	0.758	0.675	0.648	High
20	7241	UGACUCUGUGGCAAUGAGACAGACAUUCGGCAACUUGUACAAAAC	0.574	0.457	0.691	0.615	Moderate
21	7262	GACAUUCGGCAACUUGUACAAAACUUUUCACCUAAACCCAGGGAU	0.574	0.514	0.593	0.578	Low
22	7506	AACUCCCAUCACCUGUACAGGGACAAACAUUAUUUUGUGCGGGGU	0.635	0.395	0.500	0.569	Low
23	7595	GAUAAAUAACAUCAUAAUCAGGACACUCAUGCUGAAGGUGUACAA	0.597	0.574	0.521	0.565	Low
24	7722	UUGCUUGCUGAAGCUGGCAAGGACUAUGGAUUAAUCAUGACACCA	0.772	0.640	0.518	0.664	High
25	7781	GUGCUUCAAUGAAGUUACUUGGACUAACGUCACAUUCCUAAAGAG	0.730	0.673	0.579	0.666	High
26	7886	CAUACACGAAUCAAUCAGAUGGACCAAGGAUCCAAAGAAUACCCA	0.580	0.537	0.533	0.559	Low
27	8052	ACCUUACGUAGGAAGUGGUUGGACUCUUUCUAAAUUAGAGACAAU	0.721	0.688	0.549	0.650	High
28	8117	UUAACCCUACUGUACUAACCGAACUAGACAACGGUGCAGUAGGGG	0.572	0.415	0.569	0.563	Low
